# Conjunctival epidermoid carcinoma: a case report

**DOI:** 10.11604/pamj.2014.18.54.4566

**Published:** 2014-05-15

**Authors:** Adil Belmokhtar, Rajae Daoudi

**Affiliations:** 1 Université Mohammed V Souissi, Service d'Ophtalmologie A de l'Hôpital des Spécialités, Centre Hospitalier Universitaire, Rabat, Maroc

**Keywords:** epidermoid carcinoma, conjunctiva, eye

## Image in medicine

We report the case of 55 years old woman who present a conjunctival growth in the right eye (Figure A). The biopsy showed a conjunctival epidermoid carcinoma in situ. Conjunctival epidermoid carcinoma is a rare tumor, it is more common in male and elderly patients, but may occur earlier in patients with xeroderma pigmentosa or AIDS. The major risk factor for the disease is the exposure to ultraviolet light (sun light). Human papillomavirus (HPV) types 16 and 18 may also be incriminated in tumour development. The diagnosis is histological, by biopsy. The treatment depends on the locoregional and general extension, as well as health condition of the patient himself. It lies in early excision of the tumour with healthy margins followed by cryotherapy or topical chemotherapy « eye drop » using either Interferon, motomycin C or 5-Fluoruuracil. In our treatment, we used a excision with clear margin followed by topical mitomycin C (Figure B shows the conjunctival tumour after the treatment). The high rate of recurrence requires long-term follow-up.

**Figure 1:**
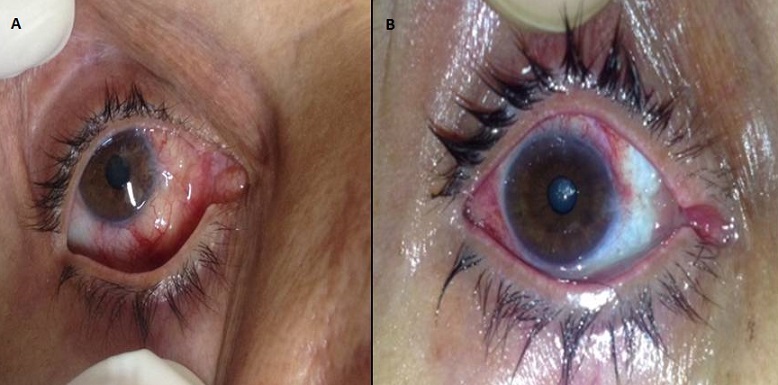
A) conjunctival growth in the right eye; B) conjunctival tumour after the treatment

